# Supplementary material for: Functional analysis of the human perivascular subarachnoid space
Source: Nat Commun. 2024 Mar 5;15:2001. doi: 10.1038/s41467-024-46329-1 (PMC10914778; doi:10.1038/s41467-024-46329-1)
Supplement: Supplementary file 1 — Supplementary Information [file 41467_2024_46329_MOESM1_ESM.pdf]

Supplementary Material

Functional analysis of the human perivascular subarachnoid space

Per Kristian Eide[1,2], Geir Ringstad[3,4]

[1]Department of Neurosurgery, Oslo University Hospital - Rikshospitalet, Pb 4950 Nydalen, N-0424 Oslo, Oslo, Norway.

[2]KG Jebsen Centre for Brain Fluid Research, Institute of Clinical Medicine, Faculty of Medicine, University of Oslo, PB 1072 Blindern, N-0316 Oslo, Oslo, Norway.

[3]Department of Radiology, Oslo University Hospital- Rikshospitalet, Pb 4950 Nydalen, N-0424 Oslo, Oslo, Norway.

[4]Department of Geriatrics and Internal medicine, Sorlandet Hospital, 4838 Arendal, Arendal, Norway.

Corresponding author:

Professor Per Kristian Eide, MD PhD

Department of Neurosurgery

Oslo University Hospital - Rikshospitalet

Pb 4950 Nydalen,

N-0424 Oslo, Norway

Email: p.k.eide@medisin.uio.no

Supplementary Figure 1

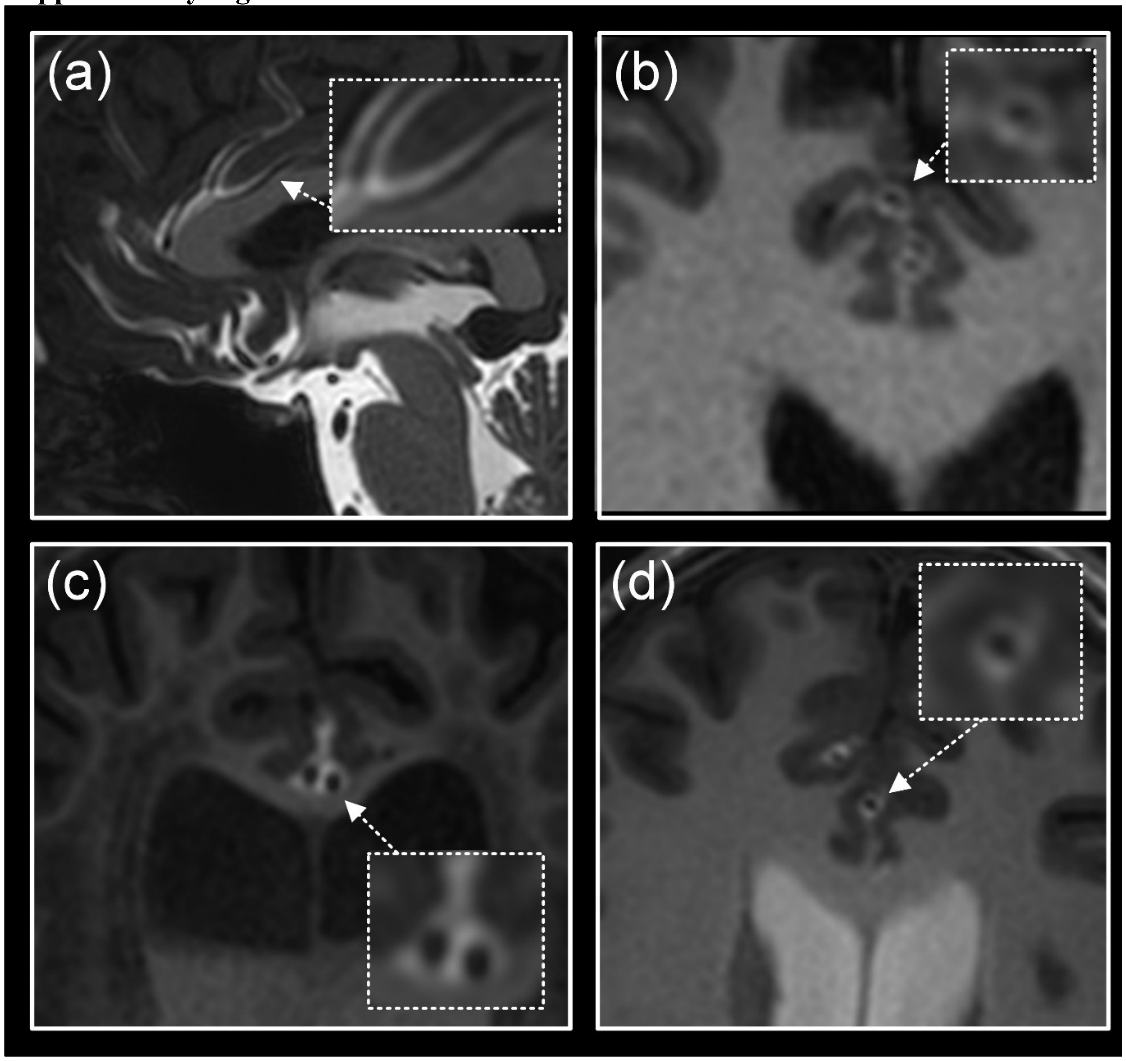

Circumferential tracer enrichment around the anterior cerebral artery (ACA). Tracer distribution occurred circumferential around the ACA (a-d). Time from intrathecal tracer injection: (a) 36 minutes, (b) 50 minutes, (c) 142 minutes, (d) 129 minutes.

Supplementary Figure 2

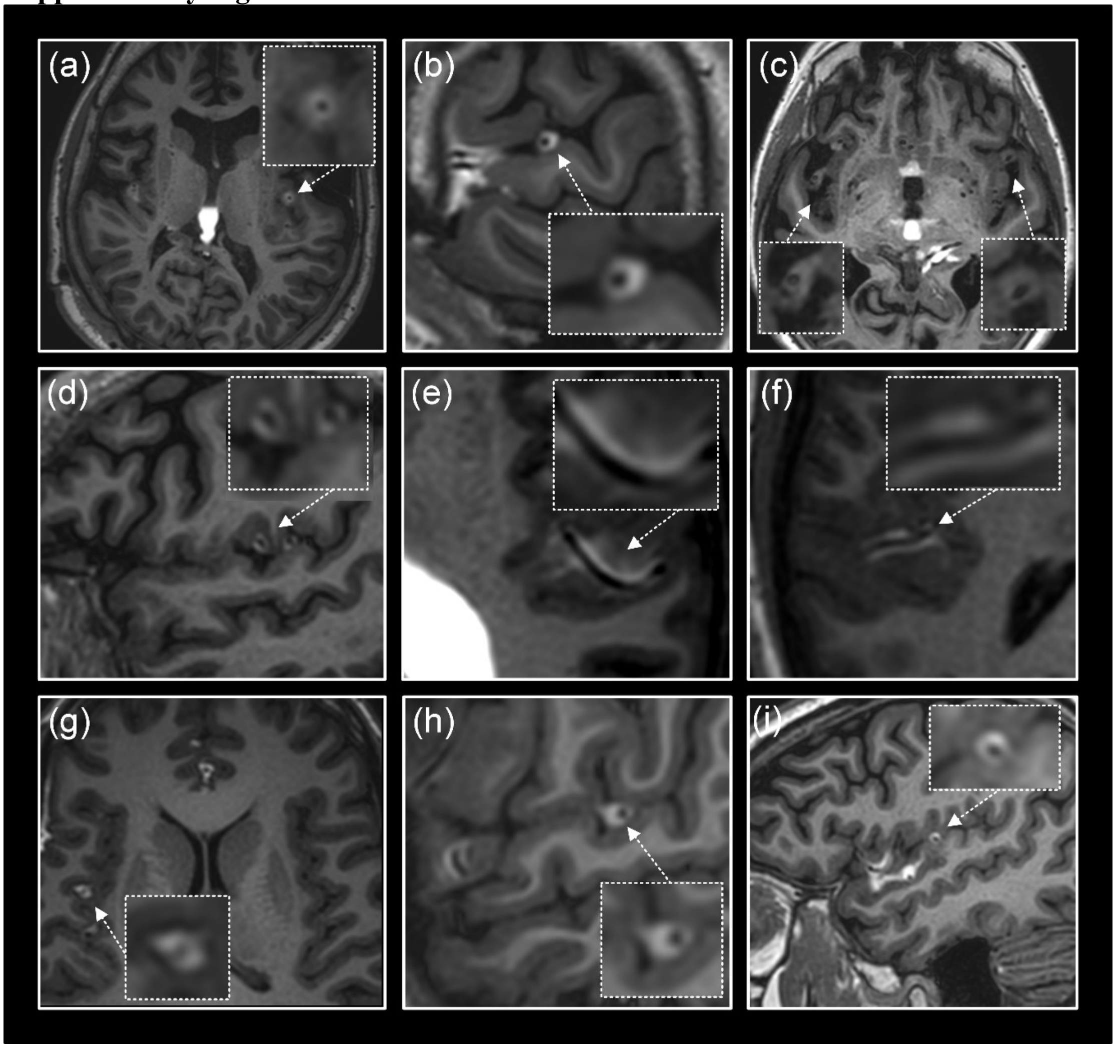

Circumferential tracer enrichment around the middle cerebral artery (MCA). Tracer distribution occurred circumferential around the MCA (a-i). Time from intrathecal tracer injection: (a) 186 minutes, (b) 149 minutes, (c) 41 minutes, (d) 31 minutes, (e) 128 minutes, (f) 47 minutes, (g) 7 minutes, (h) 42 minutes, (i) 39 minutes.

Supplementary Figure 3

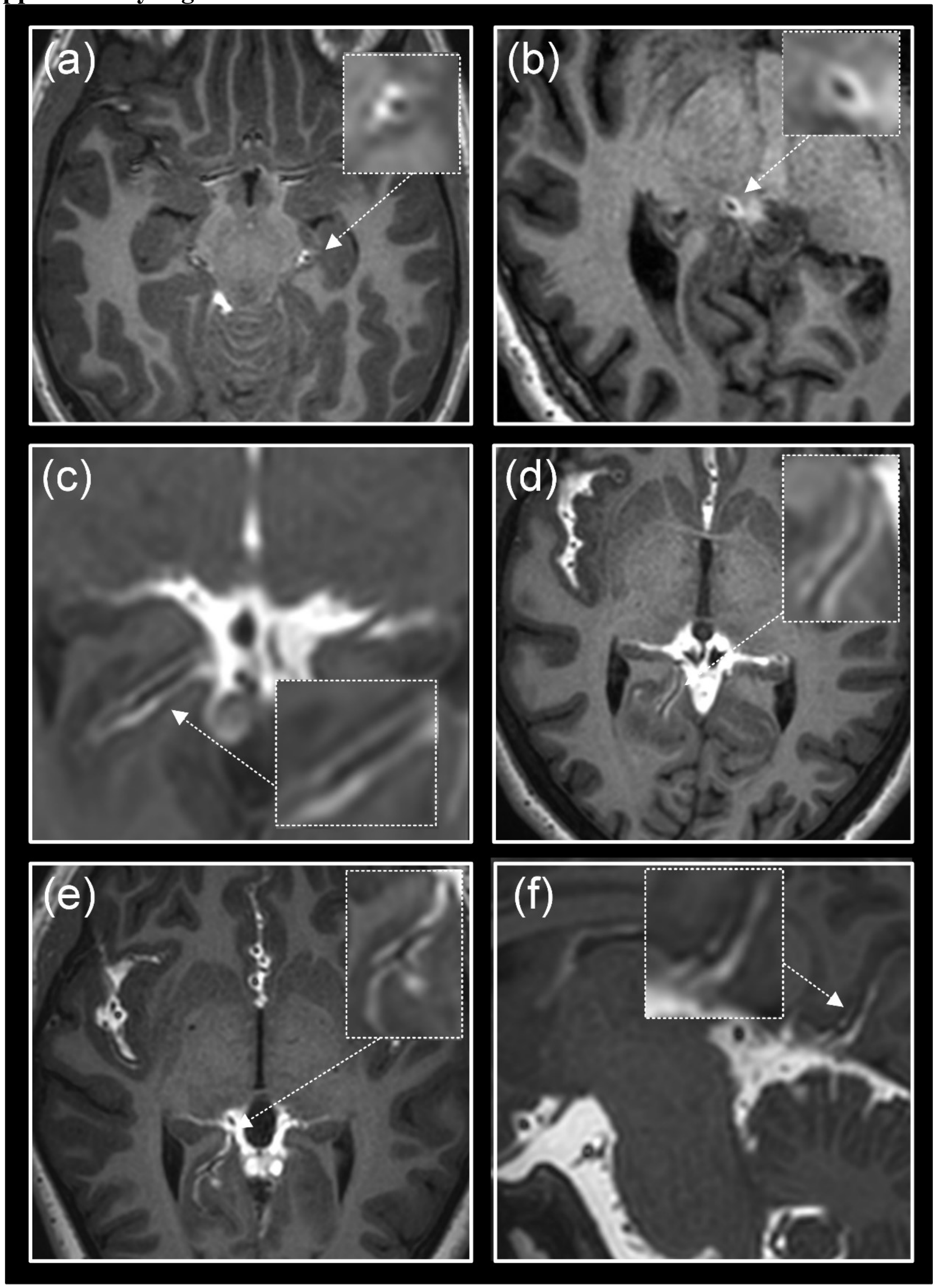

Circumferential tracer enrichment around the posterior cerebral artery (PCA). Tracer distributed occurred circumferential around the PCA (a-f). Time from intrathecal tracer injection: (a) 10 minutes, (b) 37 minutes, (c) 50 minutes, (d) 50 minutes, (e) 47 minutes, (f) 26 minutes.

Supplementary Figure 4

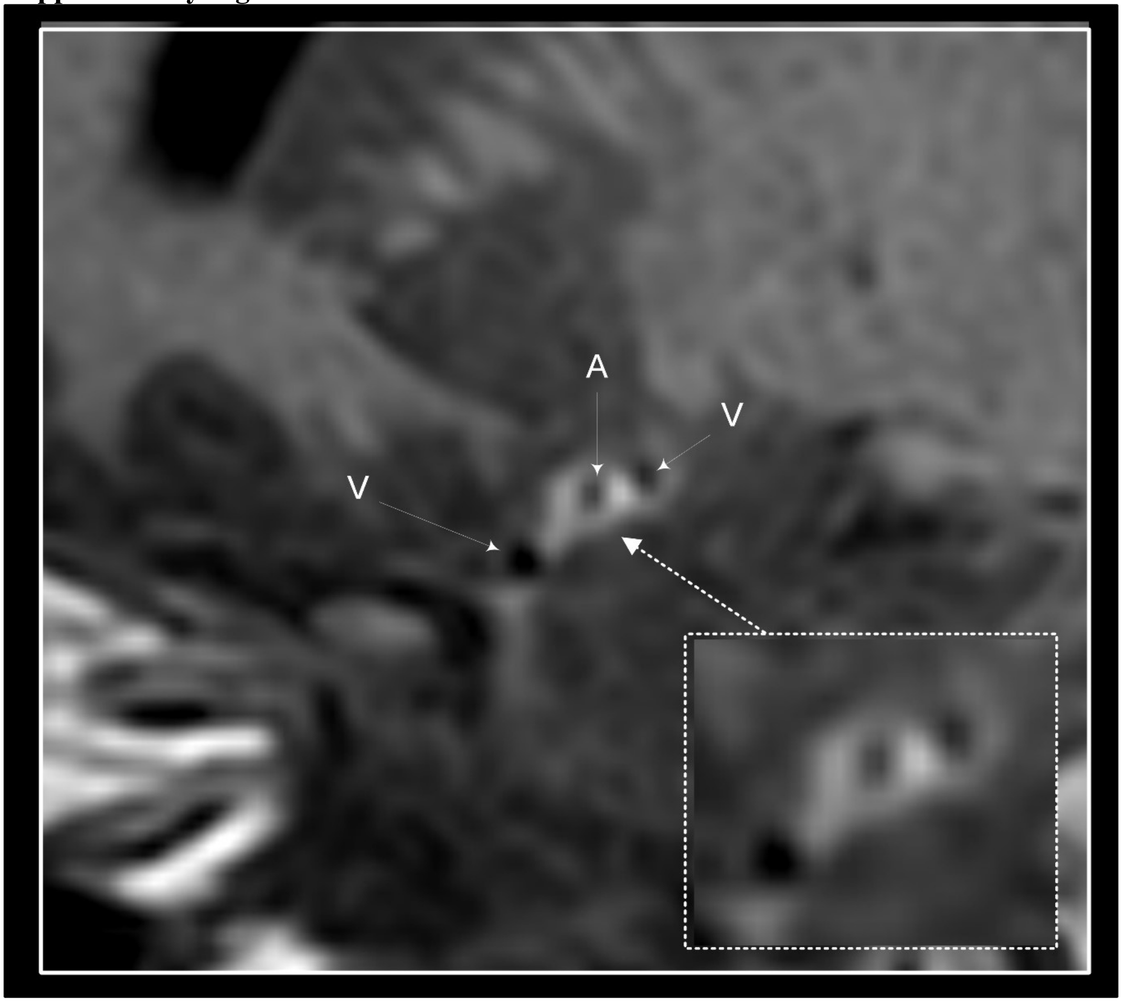

Periarterial versus perivenous tracer enrichment. Tracer distributed circumferential around the M1 segment of middle cerebral artery (MCA). Time from intrathecal tracer injection: 57 minutes. A: Artery. V: Vein.

Supplementary Figure 5

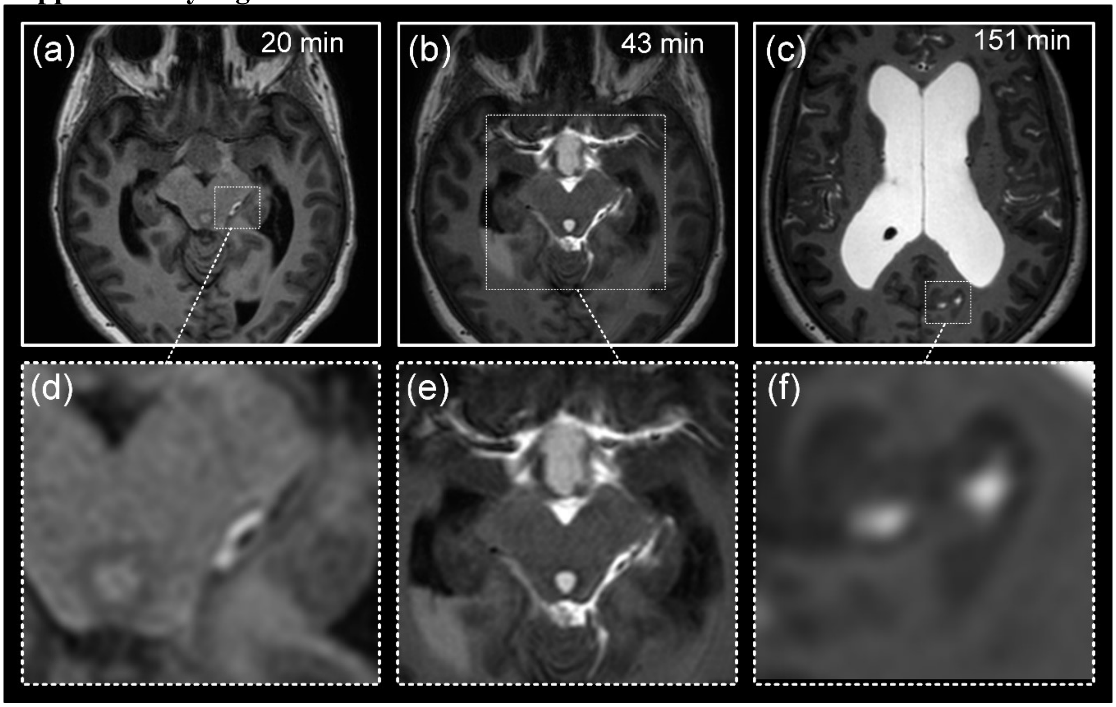

In vivo evidence for antegrade perivascular transport along the posterior cerebral artery. The time course of perivascular subarachnoid space transport along PCA (a-c) indicate antegrade tracer propagation. Magnifications of circumferential perivascular tracer enrichment are shown in (d-f). Time from intrathecal tracer injection: (a) 20 minutes, (b) 43 minutes, (c) 151 minutes. In c), also note extensive perivascular enhancement along peripheral branches of anterior- and middle cerebral arteries.

Supplementary Figure 6

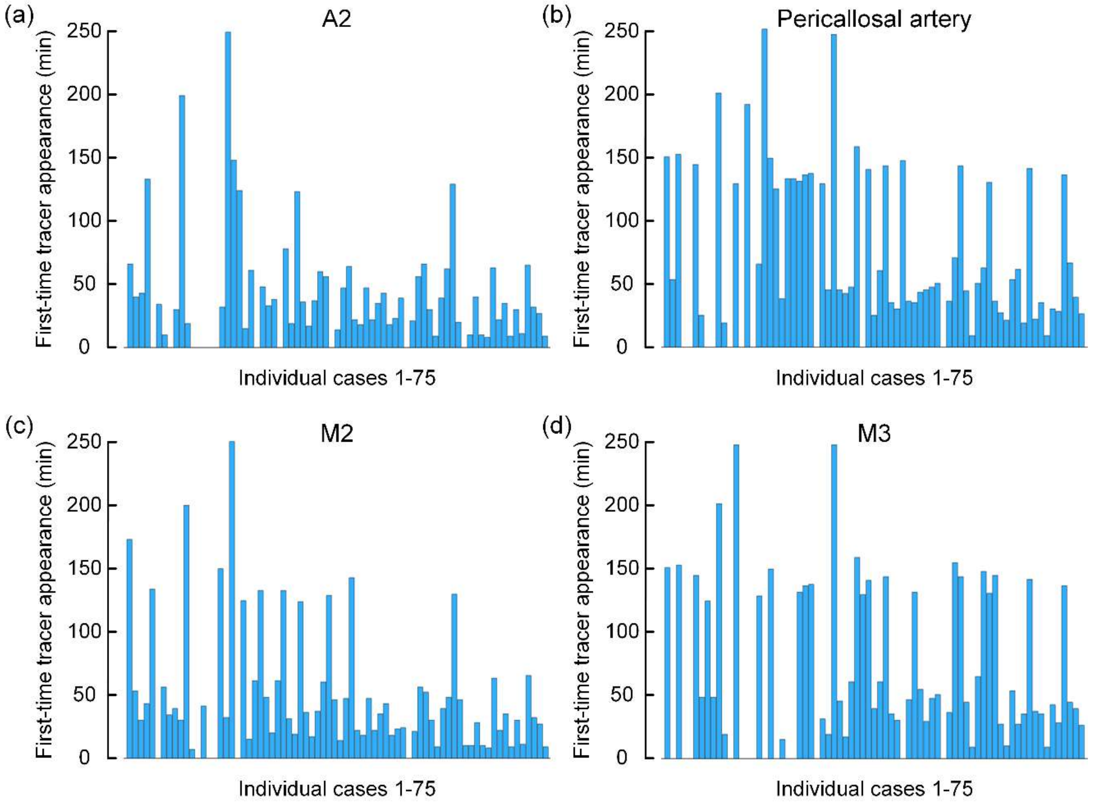

Individual variation in first-time appearance of tracer. For the individual cases included in the study, there were large variation in first-time circumferential tracer enhancement in (a) A2, (b) pericallosal artery, (c) M2, and (d) M3. Missing values represent cases where the circumferential enhancement phenomenon could not be observed in the respective vascular segment.

Supplementary Figure 7

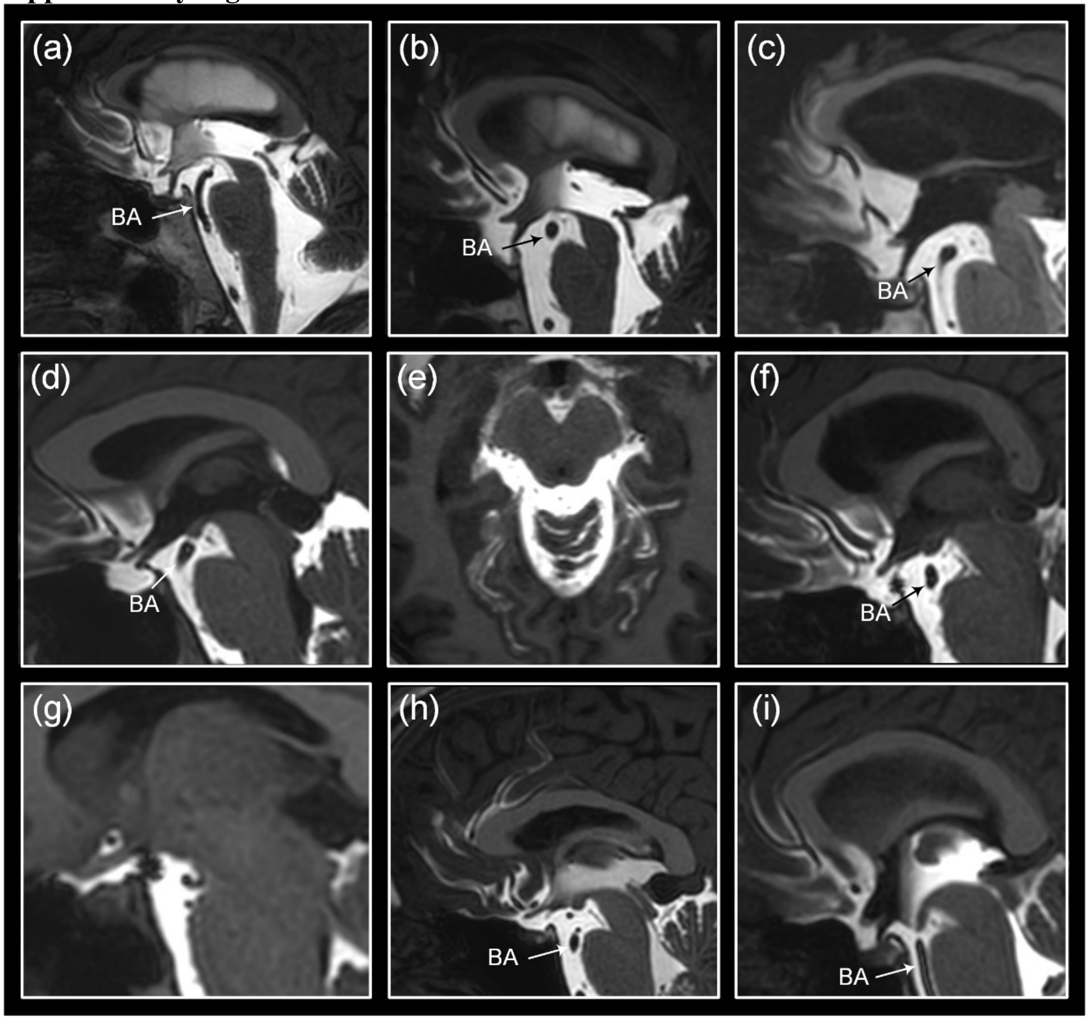

In vivo evidence for direct tracer propagation between the basal cisterns and the perivascular subarachnoid spaces. Tracer distribution indicated direct communication between basal cisterns and the perivascular compartment (a-i). Time from intrathecal tracer injection: (a) 19 minutes, (b) 45 minutes, (c) 17 minutes, (d) 23 minutes, (e) 59 minutes, (f) 14 minutes, (g) 8 minutes, (h) 36 minutes, (i) 39 minutes. Basilar artery (BA) indicated.

Supplementary Figure 8

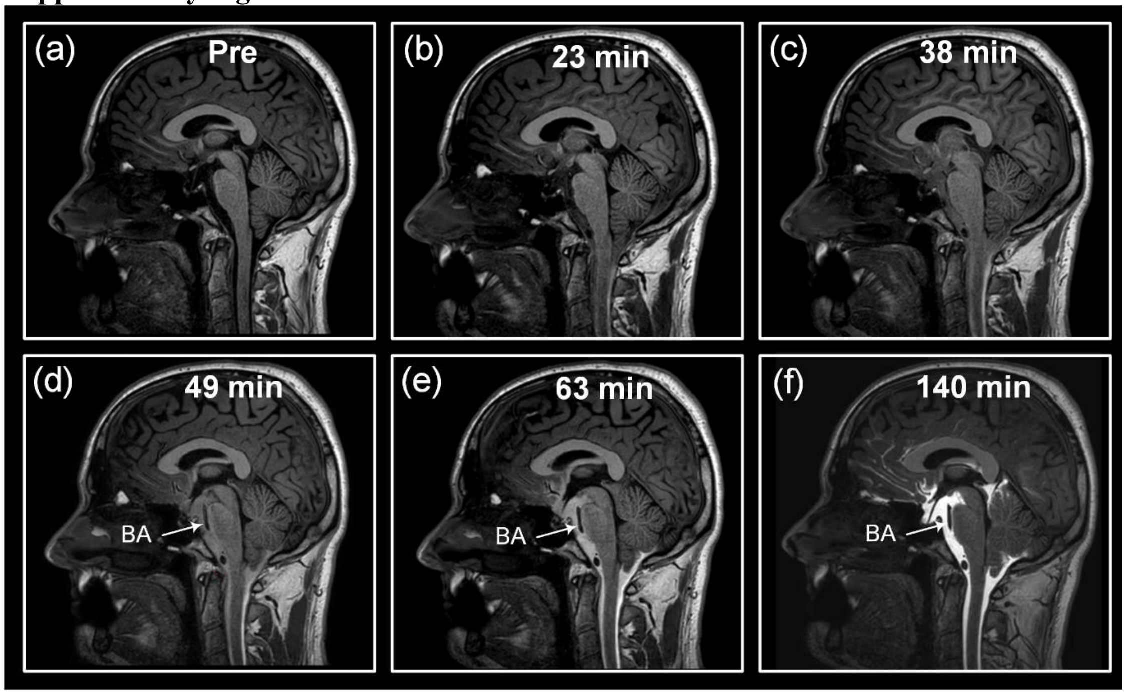

Tracer propagation between thecal sac and basal cisterns. Multiphase MRI acquisitions showing propagation of tracer from thecal sac, via basal cisterns to supratentorial compartment. Time from intrathecal tracer injection: (a) Prior to tracer injection, (b) 23 minutes, (c) 38 minutes, (d) 49 minutes, (e) 63 minutes, and (f) 140 minutes. Basilar artery (BA) indicated.

Supplementary Figure 9

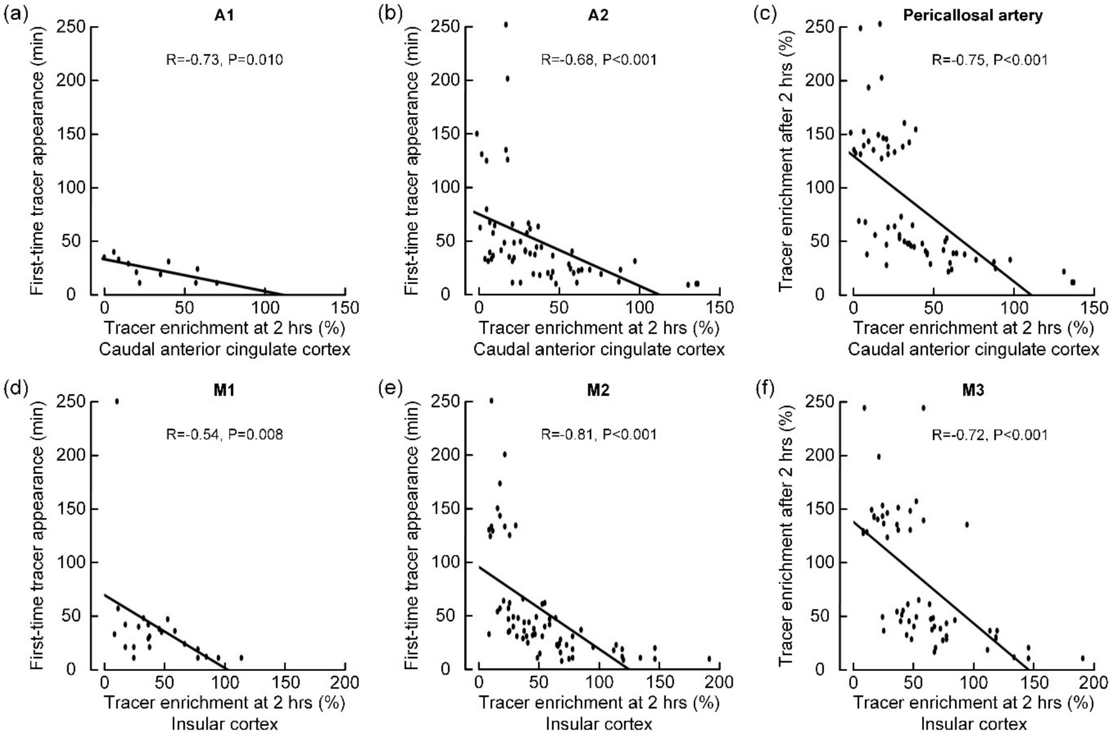

The perivascular subarachnoid tracer transport precedes the tracer enrichment in cerebral cortex. After being confined to the perivascular space, the tracer passed to the surrounding subarachnoid space (SAS) and further to the extravascular space of the cerebral cortex. (a-c) The correlation between tracer enrichment in caudal anterior cingulate cortex (gray matter) at 2 hours and first-time appearance of tracer in A1 (a), A2 (b) and pericallosal artery (c) segments of ACA. (d-f) The correlation between tracer enrichment in insular cortex (gray matter) at 2 hours and first-time appearance of periarterial tracer enhancement in M1, M2 and M3 segments of MCA. Tracer enrichment refers to percentage change in normalized t1 signal units from before contrast. For the individual plots, the Spearman correlation coefficient is given with significance level, and fit line shown.

Supplementary Figure 10

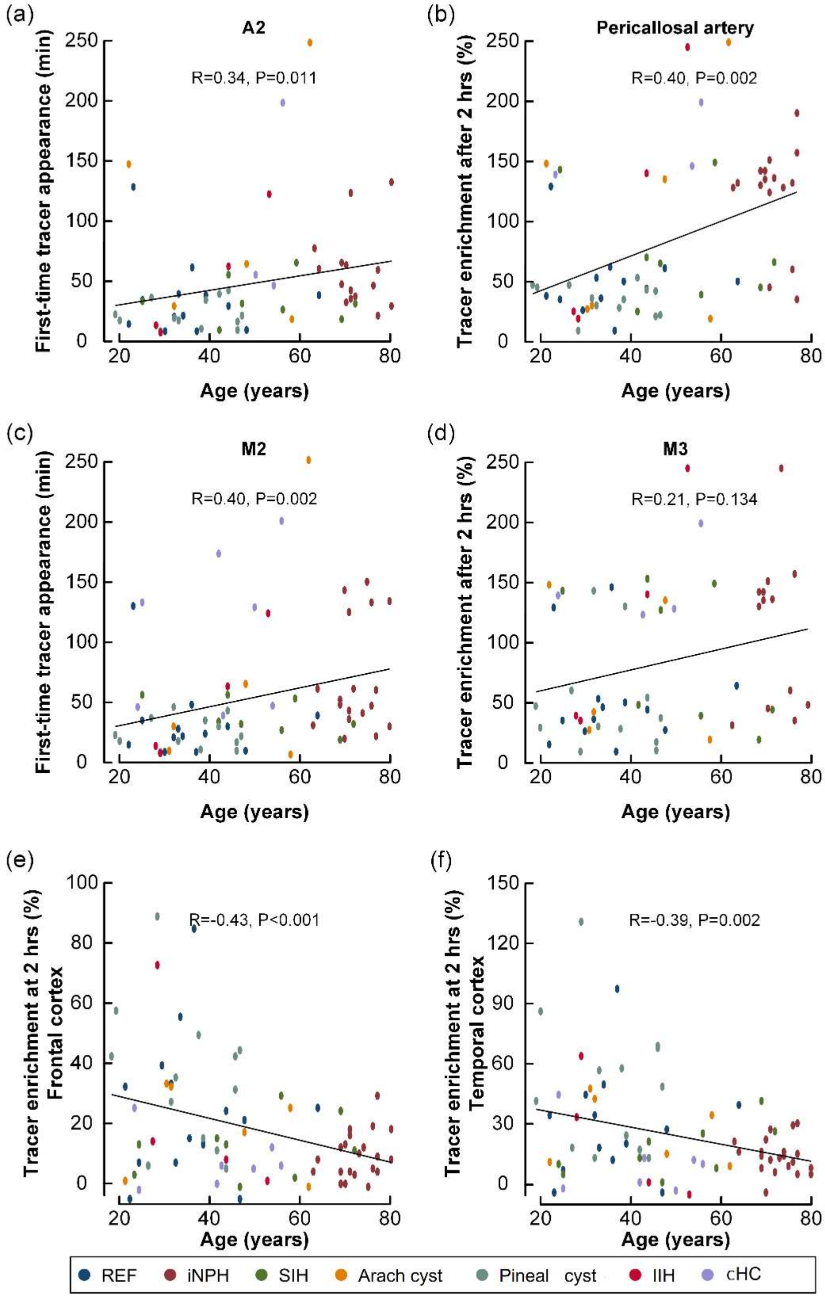

The association between periarterial subarachnoid tracer transport and age across disease categories. Considering all patient groups, the perivascular molecular transport of intrathecal tracer is slowed down with increasing age. The plots show the overall correlation coefficients between age and first-time appearance of tracer along (a) A2, (b) pericallosal artery segments of anterior cerebral artery (ACA), (c) M2 and (d) M3 segments of middle cerebral artery (MCA), and age and tracer enrichment at 2 hours in (e) frontal cortex and (f) temporal cortex (gray matter). Tracer enrichment refers to percentage change in normalized t1 signal units from before contrast. For the individual plots, the Spearman correlation coefficient is given with significance level, and fit line shown. When statistically considering the diagnosis category, the significance levels of plots became non-significant, demonstrating that disease category was a confounder.

Supplementary Figure 11

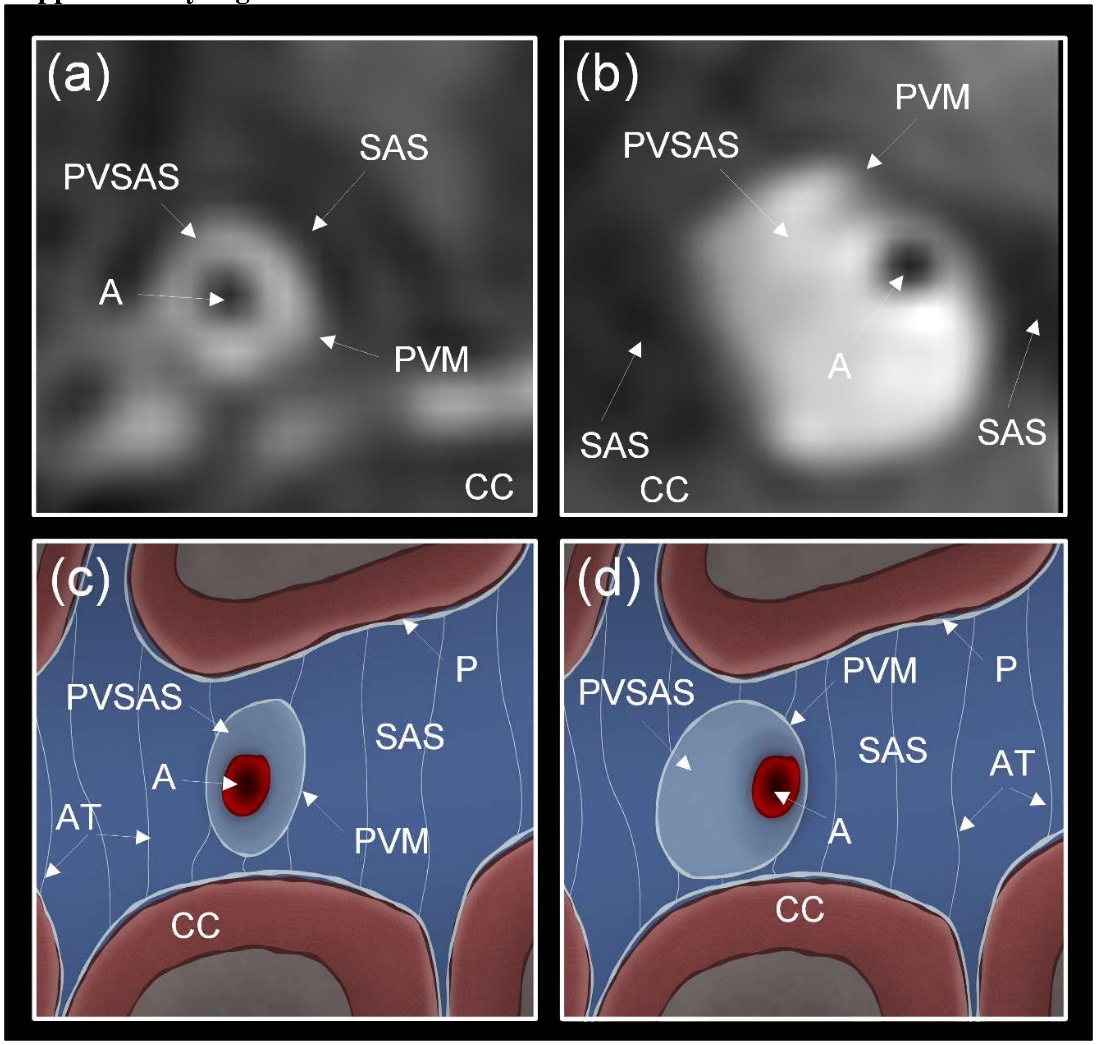

The morphology of perivascular subarachnoid space (PVSAS) is altered in the dementia subtype idiopathic normal pressure hydrocephalus (iNPH). The MR images showed periarterial tracer enrichment as a donut shaped regular structure in REF subjects (a) while in iNPH subjects the PVSAS was larger and more irregular in shape (b). A cartoon illustrates the differences between REF and iNPH subjects. A: Artery. AT: Arachnoid trabecula. CC: Cerebral cortex. P: Pia. PVM: Perivascular membrane. PVSAS: Perivascular subarachnoid space. SAS: Subarachnoid space. Illustration in (c-d): Øystein Horgmo, University of Oslo.

Supplementary Figure 12

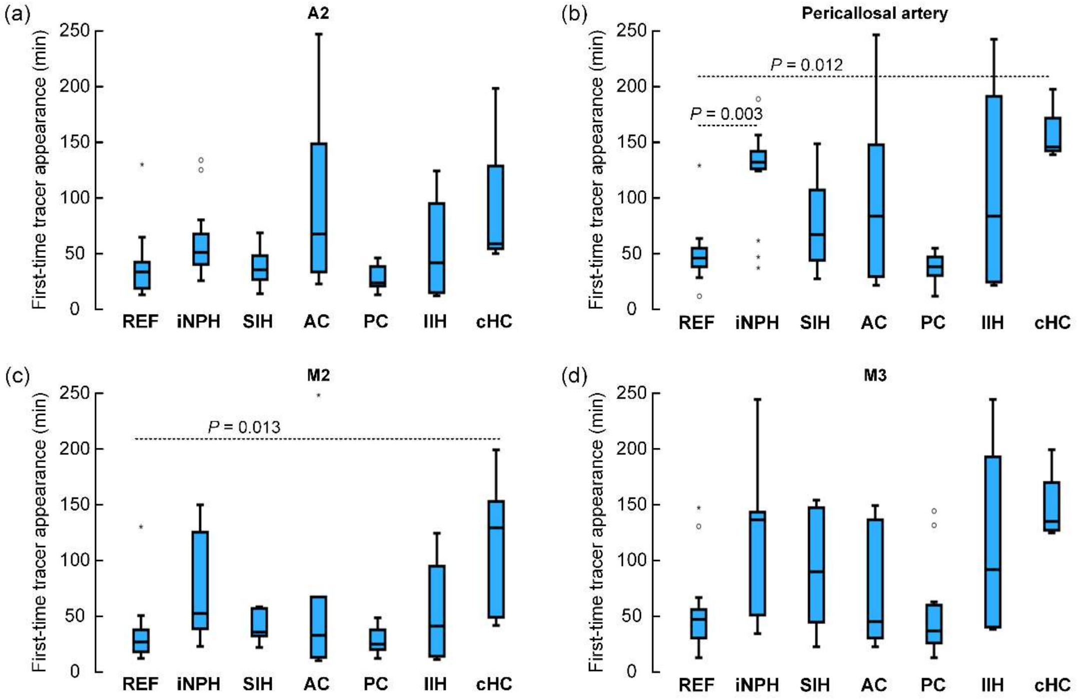

First-time appearance of tracer within the perivascular subarachnoid spaces of the anterior cerebral artery (ACA) and middle cerebral artery (MCA) depends on the underlying disease. For the diffferent patient cohorts included here, we show first-time appearance of tracer in perivascular subarachnoid spaces of the ACA segments A2 (a) and pericallosal artery (b), and the MCA segments M2 (c) and M3 (d). Box plots show median, 75% percentiles and ranges. Diagnostic catgeories: REF: Reference (n=14). iNPH: Idiopathic normal pressure hydrocephalus (n=22). SIH: Spontaneous intracranial hypotension (n=9). AC: Arachnoid cyst (n=6). PC: Pineal cyst (n=13). IIH: Idiopathic intracranial hypertension (n=4). cHC: Communicating hydrocephalus not categorized as iNPH (n=7). Differences between groups determined by analysis of variance (ANOVA) with post-hoc Bonferroni corrections to correct for multiple comparisons.

Supplementary Figure 13

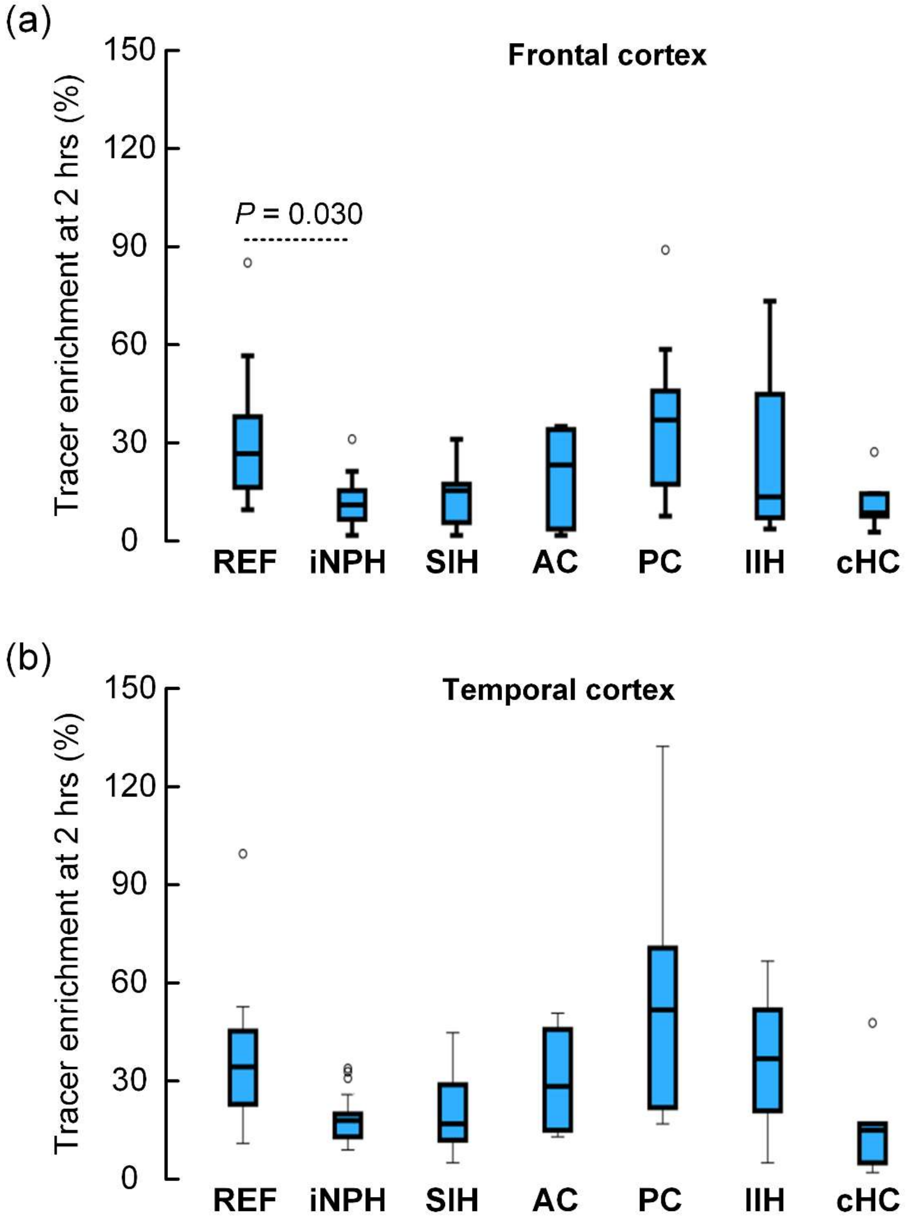

Enrichemnt of tracer in frontal and temporal cortex as related to underlying disease. For the diffferent patient cohorts included here, we show tracer enricehmnt at 2 hours in gray matter of (a) frontal cortex and (b) temporal cortex. Box plots show median, 75% percentiles and ranges. Diagnostic catgeories: REF: Reference (n=14). iNPH: Idiopathic normal pressure hydrocephalus (n=22). SIH: Spontaneous intracranial hypotension (n=9). AC: Arachnoid cyst (n=6). PC: Pineal cyst (n=13). IIH: Idiopathic intracranial hypertension (n=4). cHC: Communicating hydrocephalus not categorized as iNPH (n=7). Differences between groups determined by analysis of variance (ANOVA) with post-hoc Bonferroni corrections to correct for multiple comparisons.

Supplementary Figure 14

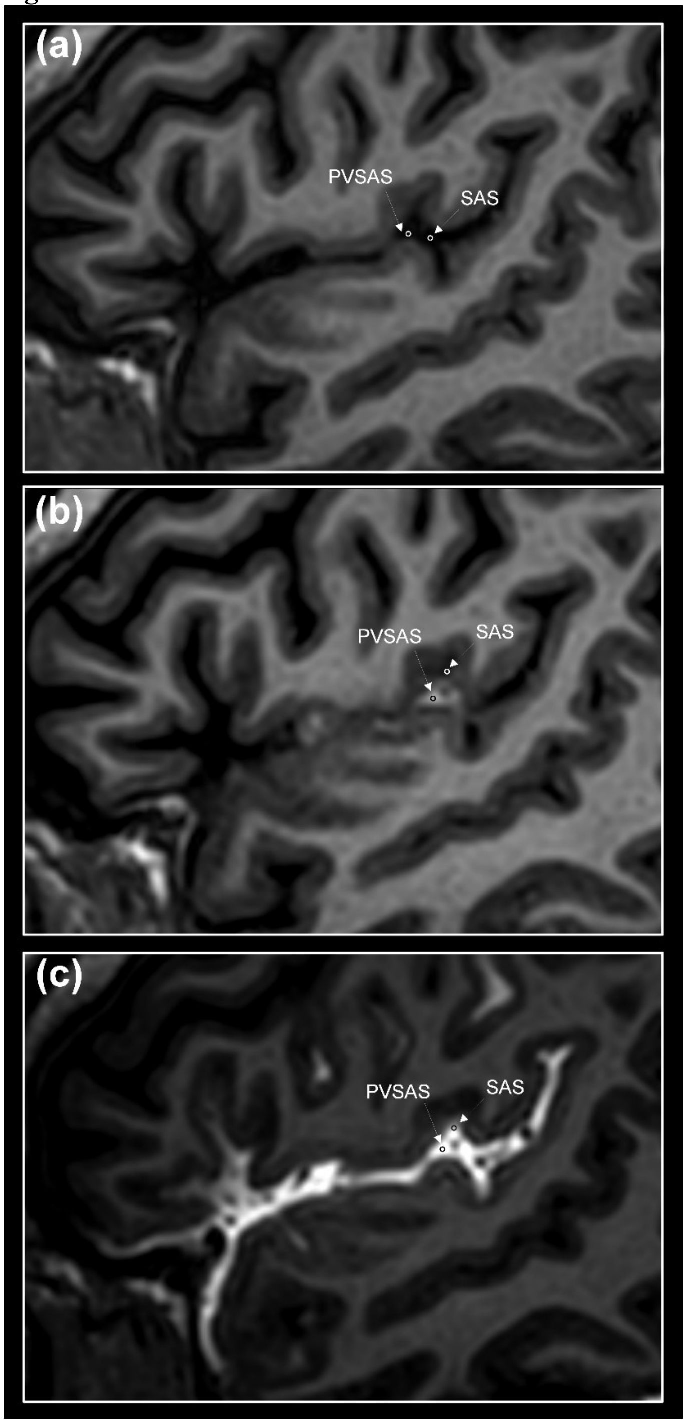

Tracer enrichment in perivascular subarachnoid space and surrounding subarachnoid space. Illustration of placement of regions of interest (ROIs) for assessment of tracer enrichment in the perivascular subarachnoid space (PVSAS) and nearby subarachnoid space (SAS). In (a) limited tracer has reached the Sylvian fissure hardly causing enrichment in PVSAS (normalized signal unit ratio: 0.05) or SAS (normalized signal unit ratio: 0.04), in (b) tracer is enriching the PVSAS (normalized signal unit ratio: 0.74) but less in SAS (normalized signal unit ratio: 0.19), while in (c) enrichment is apparent in both PVSAS (normalized signal unit ratio: 2.38) and SAS (normalized signal unit ratio: 1.71).

Supplementary Figure 15

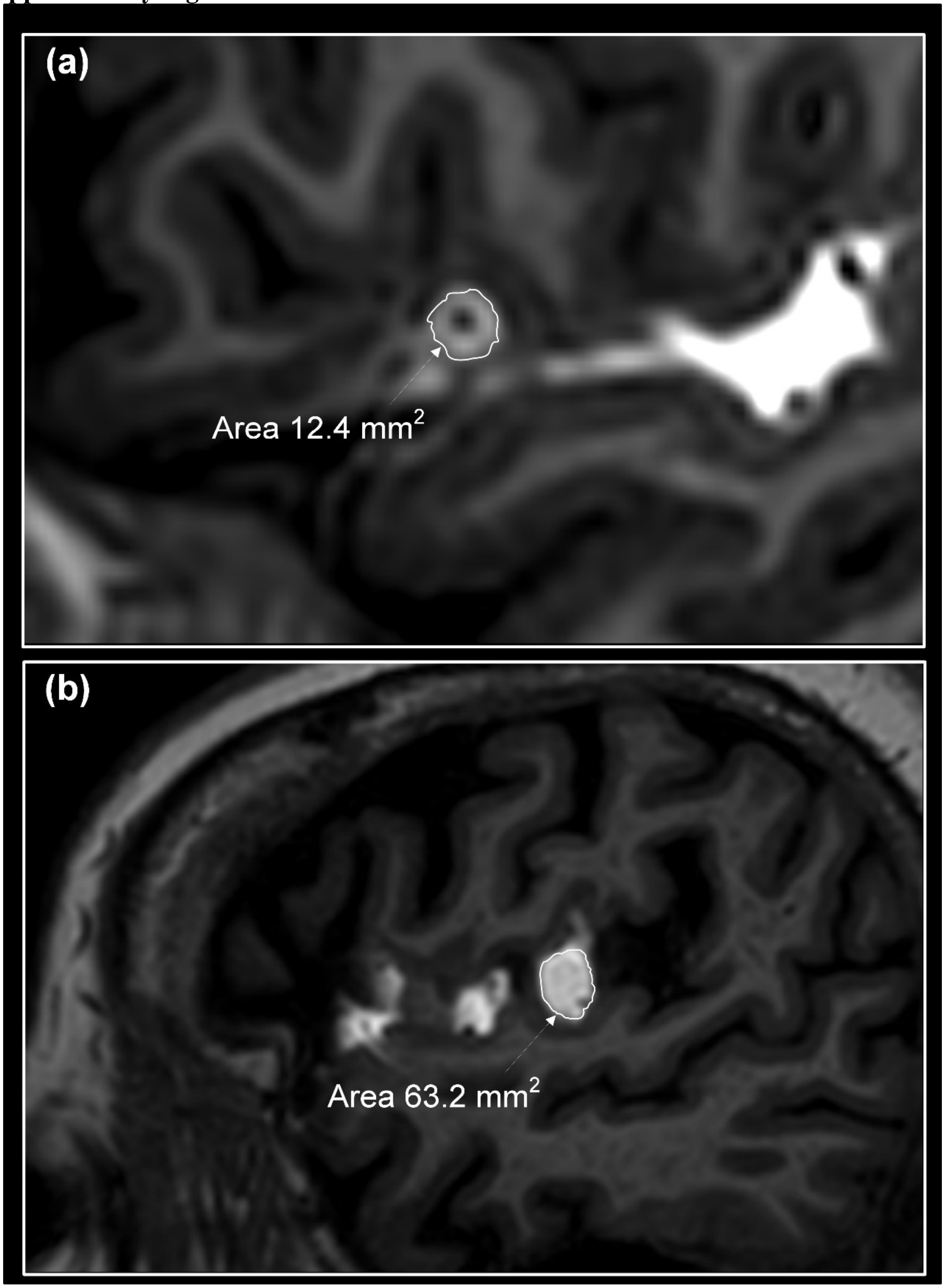

Area of perivascular subarachnoid space is enlarged in idiopathic normal pressure hydrocephalus. Methodology for estimation of area of perivascular subarachnoid space (PVSAS) in M2 segment of middle cerebral artery within the Sylvian fissure. In (a) is shown area of PVSAS in a REF subject and in (b) is shown area of PVSAS of an iNPH patient.

Supplementary Table 1. Patient groups included in the study

**Diagnosis**	
Reference subjects (REF)	14 (18.7%)
Idiopathic normal pressure hydrocephalus (iNPH)	22 (29.3%)
Spontaneous intracranial hypotension (SIH)	9 (12.0%)
Arachnoid cyst (AC)	6 (8.0%)
Pineal cyst (PC)	13 (17.3%)
Idiopathic intracranial hypertension (IIH)	4 (5.3%)
Communicating hydrocephalus (cHC)	7 (9.3%)

Data shown as numbers with percentages in parentheses.

Supplementary Table 2. Patients in each category with first tracer enrichment in subarachnoid space circumferential to an artery

	Cerebral arteries showing circumferential tracer enrichment			
**Diagnosis**	**ACA**	**MCA**	**PCA**	**VA + BA**
Reference subjects (n=14)	13 (93%)	13 (93%)	12 (86%)	0
Idiopathic normal pressure hydrocephalus (n=22)	17 (77%)	17 (77%)	10 (46%)	0
Spontaneous intracranial hypotension (N=9)	8 (89%)	9 (100%)	5 (56%)	0
Arachnoid cyst (n=6)	6 (100%)	6 (100%)	5 (83%)	0
Pineal cyst (n=13)	13 (100%)	13 (100%)	13 (100%)	0
Idiopathic intracranial hypertension (n=4)	4 (100%)	4 (100%)	3 (75%)	0
Communicating hydrocephalus (n=7)	5 (71%)	7 (100%)	4 (57%)	0
Significance	P=0.151	P=0.142	P=0.026	

Data shown as numbers with percentages in parentheses. Significance levels refer to differences between diagnoses regarding demonstration of circumferential tracer enrichment for each artery (Pearson Chi square test). ACA: Anterior cerebral artery, BA: Basilar artery, MCA: Middle cerebral artery, PCA: Posterior cerebral artery. VA: Vertebral artery.

Supplementary Table 3. Time to first circumferential tracer enrichment for the different artery segments

	First time appearance of periarterial tracer enrichment (minutes)					
**Diagnosis**	**A1**	**A2**	**Pericallosal artery**	**M1**	**M2**	**M3**
Reference subjects (n=14)	28.0	35.4 ± 32.1	48.4 ± 28.2	28.0	32.2 ± 31.4	52.3 ± 40.9
Idiopathic normal pressure hydrocephalus (n=22)	20.0 ± 14.1	58.9 ± 32.2	122.9 ± 41.2	27.0 ± 16.3	68.4 ± 45.0	112.1 ± 63.4
Spontaneous intracranial hypotension (N=9)	18.0 ± 10.5	34.5 ± 18.4	75.3 ± 46.3	20.7 ± 11.0	38.6 ± 14.3	90.3 ± 57.5
Arachnoid cyst (n=6)	34.0	102.2 ± 96.4	101.3 ± 92.2	83.5 ± 111.1	72.2 ± 101.5	74.2 ± 62.2
Pineal cyst (n=13)	20.5 ± 3.5	23.3 ± 11.7	35.2 ± 12.7	23.6 ± 7.7	24.5 ± 12.5	49.5 ± 43.7
Idiopathic intracranial hypertension (n=4)	-	52.0 ± 53.4	107.3 ± 107.4	-	52.0 ± 53.4	114.8 ± 99.5
Communicating hydrocephalus (n=7)	39.0	100.7 ± 85.3	161.3 ± 32.8	44.3 ± 9.3	109.0 ± 65.4	147.3 ± 35.1
Overall difference between groups	*P*=0.506	*P*=0.005	*P*<0.001	*P*=0.427	*P*=0.003	*P*=0.011

Continuous data given as mean ± standard deviation. Significance refers to differences between diagnoses regarding demonstration of circumferential tracer enrichment for each artery (analysis of variance (ANOVA) for overall difference between groups). A1: A1 segment of anterior cerebral artery; A2: A2 segment of anterior cerebral artery; M1: M1 segment of middle cerebral artery, M2: M2 segment of middle cerebral artery, M3: M3 segment of middle cerebral artery.

Supplementary Table 4. Tracer enrichment in cerebral cortex

	Percentage (%) tracer enrichment in gray matter two hours after intrathecal injection	
	**Frontal cortex**	**Temporal cortex**
**Total cohort (n=75)**		
**Diagnosis**		
Reference subjects (n=14)	25.6 ± 23.9	30.1 ± 25.5
Idiopathic normal pressure hydrocephalus (n=22)	10.4 ± 7.5	15.9 ± 8.3
Spontaneous intracranial hypotension (N=9)	13.1 ± 10.0	18.7 ± 12.7
Arachnoid cyst (n=6)	18.8 ± 15.0	28.3 ± 16.7
Pineal cyst (n=13)	35.8 ± 23.2	50.9 ± 33.9
Idiopathic intracranial hypertension (n=4)	24.8 ± 32.6	25.0 ± 31.5
Communicating hydrocephalus (n=7)	8.4 ± 9.0	12.7 ± 16.1
Significance	*P*<0.001	*P*<0.001

Continuous data given as mean ± standard deviation. Tracer enrichment refers to percentage change in normalized t1 signal units from before contrast. Significance refers to differences between diagnoses regarding cortical enhancement (analysis of variance (ANOVA) for overall difference between groups).
