# Supplementary material for: Functional analysis of the human perivascular subarachnoid space
Source: Nat Commun. 2024 Mar 5;15:2001. doi: 10.1038/s41467-024-46329-1 (PMC10914778; doi:10.1038/s41467-024-46329-1)
Supplement: Supplementary file 3 — Description of Additional Supplementary Files [file 41467_2024_46329_MOESM3_ESM.pdf]

<u>Description of Additional Supplementary Files</u>

Supplementary Movie 1. CSF tracer distribution in the perivascular subarachnoid space. CSF tracer propagation along the periarterial subarachnoid space of the middle cerebral artery (MCA). Movie: Tomas Sakinis, MD.

Supplementary Movie 2. Propagation of intrathecal CSF. Time-dependent propagation of tracer from thecal sac via basal cisterns to supratentorial compartment. Movie: Lars Magnus Valnes, PhD.

Supplementary Movie 3. 3D representation of intrathecal tracer distribution. The 3D representations of tracer propagation between subarachnoid basal cisterns and the perivascular subarachnoid space suggest direct passage of tracer from the thecal sac, via basal cisterns towards the perivascular subarachnoid spaces. Movie: Tomas Sakinis, MD.
