# Supplementary figures and images for: Functional analysis of the human perivascular subarachnoid space
Source: Nat Commun. 2024 Mar 5;15:2001. doi: 10.1038/s41467-024-46329-1 (PMC10914778; doi:10.1038/s41467-024-46329-1)

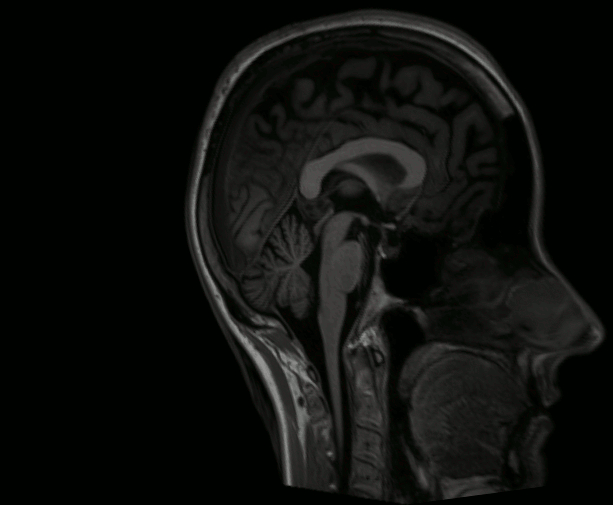

Supplement: Supplementary file 5 — Supplementary Movie 2 [file 41467_2024_46329_MOESM5_ESM.gif]
